# Observational study: effect of varying transport durations and feed withdrawal on the physiological status and health of dairy calves

**DOI:** 10.1186/s13620-025-00287-2

**Published:** 2025-01-13

**Authors:** Luca L. van Dijk, Susanne Siegmann, Niamh L. Field, Katie Sugrue, Cornelis G. van Reenen, Eddie A. M. Bokkers, Muireann Conneely, Gearoid Sayers

**Affiliations:** 1 Teagasc, Animal & Grassland Research and Innovation Centre, Moorepark, Fermoy, Ireland; 2 Department of Biological and Pharmaceutical Sciences, Munster Technological University Kerry, Tralee, V92 CX88 Ireland; 3 Animal Production Systems group, Wageningen University & Research, P.O. Box 338, Wageningen, 6700 AH The Netherlands

**Keywords:** Animal welfare, Journey duration, Feed deprivation, Hunger, Dehydration

## Abstract

Long-distance transport and associated fasting of unweaned calves have the potential to compromise the animals’ welfare. This observational study aimed to determine how transport and fasting durations impacted the physiology and health of 115 transported calves in three transport groups; IRE (*n* = 20, mean age 29.8d; short road transport (~ 29 h incl. resting time) and short feed deprivation (~ 11 h)), INT (*n* = 65, mean age 24.9d; long road/ferry transport (~ 79 h incl. resting times) and long feed deprivation (~ 28 h and 25 h)), and NLD (*n* = 30, mean age 17.7d; short road transport (~ 28 h incl. resting time) and long feed deprivation (> 18 h)). All calves travelled through an assembly centre. Each calf was blood sampled (arrival at destination farm, 1-week and 3-weeks post-arrival), health scored (arrival, 1, 3, 7, 8, 20d post-arrival) and weighed (farm/mart of origin [IRE and INT only], arrival, and 3-weeks post-arrival). (Generalised) linear mixed models were used to analyse differences in blood variables, weight, and health scores on arrival and during recovery (all other timepoints). Despite differing transport durations, both INT and NLD calves exhibited glucose, beta-hydroxy-butyrate, non-esterified-fatty-acids and sodium levels outside reference limits upon arrival, which were different from values observed in IRE calves (*p* < 0.05). Lactate and potassium were above reference range for INT calves on arrival, and higher than in IRE and NLD groups (*p* < 0.05). One- and three-weeks post arrival, most variables returned to within reference ranges, and differences between groups were minimal and not clearly associated with either transport duration or fasting during transport. Health scores did not differ between transport groups at arrival, and differences were minimal during the three-week recovery period. INT calves lost more weight during the journey than IRE calves (*p* < 0.01), while INT and NLD calves gained similar weight in the 3-weeks post-arrival, but less than IRE calves (both *p* < 0.01). Overall, changes in the physiological status of calves post transport appeared to relate more to the duration of feed deprivation than to the duration of transport, except for potassium and lactate (muscle fatigue), which were impacted more for INT calves. Most variables showed clear signs of recovery to within reference levels for all groups within three weeks. Minimizing the duration of feed deprivation during transport should be a key consideration for the dairy industry to reduce the impact of transport on calf welfare.

## Introduction

Long distance transport of unweaned, ‘non-replacement’ calves coming from dairy farms represents a focal point of physical and biological challenges which could compromise the animals’ welfare [1, 2]. The impact of altered physiological status and health of calves during and after transport has been well documented and has been shown to be triggered by a combination of factors including a reduction in feed and water intake, and increased physiological and physical stressors, muscle fatigue and immunosuppression [3–9]. The culmination of these cofactors, albeit with unequal contributions, can lead to an increased likelihood of altered physiological status and health [10–12]. The amount of antimicrobials is higher in calves that have been bought through livestock marts and when calves from multiple sources are amalgamated on a veal farm [13], both of which are common factors in veal systems.

Within Europe, transport of calves is rarely a singular event but rather a combination of stops at livestock markets, assembly centres and/or control posts, intermingled with periods of truck transport by road, and in some instances, by sea. Duration of the journey, therefore, is a combination of some or all of these movement events, leading to extended or repeated episodes of transport and feed withdrawal, even when operating within legal requirements [14].

While international transport is likely to be of greater distance and duration, prolonged feed deprivation can apply to both national and international transports. For example, the total journey duration of Irish calves transported internationally to the Netherlands is approximately 79 h [15]; incorporating one sea and multiple road transports, with a stop in an Irish assembly centre where calves are collected and fed prior to transport, and a stop in a French assembly centre where calves are fed and rested. In the Netherlands, nationally transported calves are transported from their dairy farm of origin to an assembly centre where they are fed and rested, and can remain there for up to 30 h (NL regulation #WJZ/18205482) before being transported to a regional veal farm, which may take no more than a few hours by road. By means of comparison to this system, Irish calves transported nationally to be reared for beef can have a journey time of approximately 12 h when sold through a livestock mart (a location where calves from many different locations are sold through an auction process), or approximately 6 h if sold from farm of origin; they are not typically fed until they arrive at their final destination.

Because of this journey variability, few studies have tried to pinpoint the factors that contribute most to increased morbidity and compromised homeostasis. Previous research has indicated that the physiological status of calves transported for longer distances is impacted to a greater degree [12], particularly in terms of energy balance, dehydration and muscle fatigue. Blood parameters related to energy balance such as blood glucose, beta-hydroxy butyrate (BHB), and non-esterified fatty acids (NEFA) often normalise within 24 h after 6 to 18 h of travel [11]. However, indicators of dehydration such as blood urea and lactate display more variability and may gradually return to normal levels over a span of one to five weeks post-arrival [4, 11].

Aside from changes in the energy balance and dehydration status of calves during transport, elevated cortisol levels, indicative of physical strain, have been found in calves transported for greater durations (18 h vs. 6 h), while navel inflammation was observed more frequently in calves transported for 6 h vs. 18 h [10, 11]. Furthermore, an increased duration of transport has been shown to increase mortality rates during transport in young calves (approximately four to seven days of age) transported to slaughterhouses in Australia and New Zealand [16, 17]. For this reason, transport of calves of this age is not allowed within the European Union.

Differentiating the key drivers of altered physiological status and health in transported calves (either transport duration or feed withdrawal), is essential for the development of interventions that improve the welfare of calves during transport. Therefore, the purpose of this observational study was to determine how transport and fasting durations impacted the physiology and health of transported calves in three transport groups. In this instance, we compared Irish calves destined for an Irish beef system, Irish calves destined for a veal farm in The Netherlands, and Dutch calves destined for the same veal farm in The Netherlands. We hypothesised that longer durations of fasting and transport would lead to greater physiological disturbances in unweaned calves post transport.

## Materials and methods

### Calf selection

Data for this prospective observational study was taken from three groups of calves (a) those transported nationally within Ireland (IRE), (b) those transported internationally from Ireland to the Netherlands (INT), and (c) those transported nationally within The Netherlands (NLD). The IRE and INT groups were assessed across two journeys in April and May of 2022, while the NLD group was assessed across one journey in April 2022. The IRE group was deemed to have short transport and short fasting durations, the INT group had long transport and long fasting durations, and the NLD group had short transport but long fasting durations.

Minimum sample size was set at 20 calves per group based on an initial sample size calculation using means for glucose of 3.4 (raw data from an earlier trial following calves on a commercial transport from Ireland to France) and 4.4 mmol/L (mean value for non-fasted calves [18]) and an expected standard deviation of 1.0 mmol/L. Using a power of 0.8 this calculation yielded an effective sample size of 34 calves (*n* = 16 per group), to allow for dropouts, 20 calves was set as the minimum number of animals required per group. Because of the novelty of this analysis and uncertainty in expected physiological expectations, sample collection for 30 calves per group was approved by ethical commissions to ensure valuable outcomes. The INT group was part of a larger study assessing calves during long-distance transport, which resulted in a larger group size.

The selection criteria for enrolling calves in the study were as follows: for both the IRE and INT groups, calves were enrolled into the study from eight commercial dairy farms (Co. Cork, Ireland), who routinely sold calves to a collaborating exporter. When visited one day prior to transport in April or May 2022, all available calves eligible for export on each farm were enrolled in the trial. Calves were eligible for export when they were between 14 and 40 days of age, weighed > 40 kg, and were dairy or dairy X beef breed, Jersey bred calves were excluded, every animal was assessed by a veterinarian (Department of Agriculture Food and the Marine) at the assembly centre and calves deemed unfit for transport were excluded. Fitness for transport was not assessed by the research team as this study was observational and followed commercial transports. On dairy farms, calves were randomly assigned to either the IRE (*n* = 20) or INT (*n* = 27) group. As calf details (age, breed, and sex) were unknown to the study team until their arrival at each farm, group assignments (IRE or INT) were made sequentially on a farm-by-farm basis. The trial team balanced calves between treatment groups, optimizing the distribution as equally as possible across transport groups. Age limits were set by the purchasing veal company. On all farms, at least one calf was enrolled in the IRE group and two calves in the INT group. Additionally, two commercial marts (Co. Cork, Ireland) were visited in April or May 2022, from which 39 additional calves purchased by the commercial livestock exporter were enrolled into the INT group only. Calves (*n* = 30) for the NLD group were selected based on being born at Dutch dairy farms, they were between 14 and 42 days of age and were selected at the assembly centre by a Dutch veal company.

### Transport

**Fig. 1:**
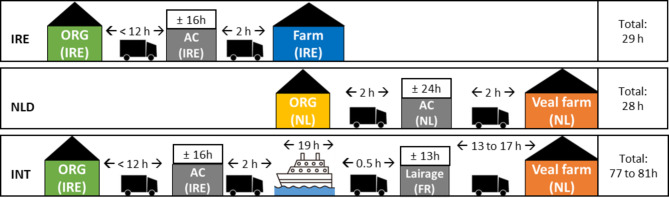
Journey layout for short- (IRE & NLD) and long-distance transport (INT) calves, including the location of origin (ORG), assembly centre (AC), lairage, destination farm or veal farm, and approximate transport durations between locations and approximate total journey times per group. Transport of NLD calves is shown, but calves were first sampled on arrival at the destination farm and not prior to this point. Locations shown are in Ireland (IRE), France (FR), or The Netherlands (NL)

Transport times (time spent on the truck) and journey times (departure from farm/mart of origin to arrival at veal farm) are described below. A layout of the transport journey for all groups, including estimated transport and journey times, is shown in Fig. 1. Not all transport sections were tracked in detail and some transport, resting, and fasting times were estimated. All transport events conformed to national and European transport legislations for stocking density (0.30–0.40 m2 per calf; EC 1/2005), lorries were fitted with deeply bedded straw. Loading conditions were assessed and approved by department veterinarians prior departure. Calves for both the IRE and INT groups, across both April and May cohorts, were transported for < 12 h (likely between 6 and 12 h, but this section of the journey was not tracked) from the dairy farm or livestock mart of origin to an assembly centre (Leinster, Ireland), arriving about midnight and were offloaded to straw pens (maximum 20 calves per pen, dimensions approx. 5 × 5 m), they remained at the assembly centre for 12 to 16 h. Calves arrived on many different trucks from different locations which were unable to be tracked by the trial team and arrival times are not recorded at the assembly centre. Calves were fed 2 L of milk replacer [125 g/L fed at 40 °C; 21% protein, 17% fat] in teat feeders the following morning (approximately 8 h after arrival). That afternoon, IRE calves were loaded onto a commercial livestock lorry and transported for approximately 2 h to their destination (Teagasc Grange Research Centre, Dunsany, Co. Meath, Ireland) where they were offloaded and penned. At the same time, INT calves were loaded onto a three-tier commercial lorry and transported for approximately 1 h by road to a ferry port in Dublin, Ireland (April cohort) or Rosslare, Ireland (May cohort), where the lorry boarded a ferry to Cherbourg, France for a 18 to 20 h sea journey. On arrival in France, the lorry drove 30 min to a lairage (Normandy, France) where the calves were offloaded and fed 3 L of milk replacer [90 g/L fed at 40 °C; 22% protein, 19% fat] in teat feeders and rested on straw (average of 40 calves per pen) for 13 h. Upon re-loading, calves were transported by road for an additional 13 h (April cohort) or 17 h (May cohort) to the destination veal farm (two separate farms for April and May cohorts in the province of Gelderland, The Netherlands). On arrival, calves were offloaded and penned individually at a stocking density of 1.6m2 per calf (pen dimensions typically 1.6 × 1.0 m). NLD calves were transported from Dutch dairy farms of unknown origin to an assembly centre in The Netherlands and remained there for an unknown duration, but not greater than 30 h and commonly less than 24 h (personal communication). The feeding regime at this centre was unknown. Calves were loaded onto a lorry at the assembly centre and transported to the same veal farm as the INT April 2022 cohort. They arrived three days after the INT group, and on arrival were offloaded and penned individually.

The total journey time for IRE calves was approximately 29 h and they were fasted for approximately 11 h before sampling on arrival at their destination farm. The total journey time for INT calves was 77 (April cohort) to 81 (May cohort) hours and they were fasted for approximately 27 to 28 h between Ireland and France, and for approximately 23 to 27 h between France and arrival at the destination farm in The Netherlands. The total journey time for NLD calves was estimated at 28 h and, based on energy balance and dehydration indicators in blood, were retrospectively estimated to have been fasted for > 18 h before arrival at the destination farm.

### Husbandry procedures after arrival at destination farm

Husbandry procedures, in particular housing and feeding regimes, differed between the IRE group versus the INT and NLD groups. IRE calves were housed with 10 calves per pen on deeply bedded straw and were fed 3 L of milk replacer [125 g/L fed at 40 °C; 26% protein, 16% fat] twice a day in teat feeders. Calves of the INT and NLD groups were randomly housed in individual wooden slat pens according to Dutch housing legislation for veal calves for at least 3 weeks post-arrival. Calves were separated by stainless steel fencing but were able to see and touch calves in neighbouring pens. INT and NLD calves were fed an electrolyte mix in 2 L of water for the first feed post arrival (as part of the standard on-farm protocol), from the next feeding onwards calves were fed 1.5 L of milk replacer [130 g/L mixed at 45 °C; 21% protein, 18.2% fat] twice a day in buckets, the volume gradually increased to 2.7 L twice a day by 3 weeks post arrival.

During the April transport cohort for IRE and INT calves, weather was mild with a slight overcast (15.8 °C, humidity: 72%; average data recorded by two TinyTags positioned on the inside wall in the front and rear of the INT lorry; temperatures for IRE calves were between 5.4 °C and 15.5 °C (Phoenix park weather station, Met Éireann), for INT calves the sea was calm with minimal swell [mean wave height of 1.6 m at M5 buoy; [19]]. For INT calves transported for the second day between France and the Netherlands, the weather was mild with a slight overcast (15.9 °C, humidity: 67%). For the May transport cohort for IRE and INT calves, the weather was mild with a slight overcast (17.7 °C, humidity: 66%; average data recorded by two TinyTags positioned on the INT lorry; temperatures for IRE calves were between 9.7 °C and 17.2 °C (Phoenix park weather station, Met Éireann), for INT calves the sea was rough [mean wave height of 3.6 m at M5 buoy; [19]]. For INT calves transported for the second day between France and the Netherlands, there were heavy downpours (20.3 °C, humidity: 82%; average data recorded by two TinyTags positioned on the inside wall in the front and rear of the INT lorry). Transports of NLD calves were not tracked and no lorry temperature details were collected, weather reports reported temperatures between 2.4 °C and 13.8 °C on the day of transport (de Bilt weather station, KNMI). Barn temperature in Ireland and the Netherlands was inconsistently recorded, but no unseasonable variations in weather events occurred during the study.

For INT and NLD calves, blanket antibiotic treatments were prescribed by a veterinarian based on a criterion of 10% of calves with disease within a five-day period or 4% of calves newly diagnosed with disease within 24 h. Individual treatments, where applicable, were established jointly by the farmer and veterinarian and were based on external signs of disease, unwillingness to finish their feed allowance and altered rectal temperature.

### Sampling

All calves were blood sampled on arrival at the destination farm (Arrival), 7 to 10 days post arrival (1WK), and 17 to 21 days post arrival at the destination farm (3WK). Calves were fed 3 to 9 h before sampling at the 1WK and 3WK time-points.

Weighing of calves was performed at the point of origin for IRE and INT calves only, while all calves were weighed on arrival and three weeks post arrival using calibrated weighing scales (Sampling in Ireland: EziWeigh 7i Cattle Weighing Scales; sampling in the Netherlands: Calf scales Type ISC-V, Henk Maas Weegschalen B.V, Veen, NL). Differences in body weight over time (delta weight) were calculated between origin and arrival (IRE and INT only), and between arrival and 3 weeks post arrival (all three groups).

Additionally, an external clinical health scoring system based on the Wisconsin calf health score [20], and research by Barry et al. [21] and Marcato et al. [11] adapted for use in transport situations, was employed to assess the health of calves on arrival and 1, 3, 7, 8, and 20 days post arrival. Health scoring criteria were scored in a 0–3 tier system, but were transformed to binary variables (0 = healthy, 1 = not healthy) prior to analysis due to low numbers of some ordinal categories for some health variables. Binary health scoring criteria are presented in Table 1. Health scoring was performed by five different observers, observers in the Netherlands reached 75–87% inter-rater agreement. Inter-rater agreement was not assessed between observers in Ireland and the Netherlands, as observers being located in different countries made inter-rater agreement testing logistically impossible. However, all observers met online multiple times prior to the study and discussed the scoring system with the aid of photographic examples to ensure agreement and clarity on scoring criteria.

**Table 1:** Health scoring criteria used to assess external, clinical health of calves (based on Wisconsin calf health protocol [20] and research by Barry et al. [21] and Marcato et al. [11])

Indicator	Score	Criteria
Eye discharge	0	No discharge
	1	Small or moderate amount of unilateral or bilateral ocular discharge
Altered ear	0	Normal ear position or slight unilateral droop
position	1	Head tilt or bilateral droop
Nose discharge	0	Normal serous discharge
	1	Small amount of unilateral cloudy discharge, moderate amount of bilateral cloudy discharge, or copious bilateral mucopurulent discharge
Coughing	0	No cough
	1	Occasional or continuous cough
Skin tent (> 1s)	0 1	Normal, skin tent returns to normal < 1s Dehydrated, skin tent returns to normal > 1s
Navel	0	Normal (up to approx. width of pointer finger)
inflammation	1	Mild (more than approx. width of pointer finger), or moderately inflamed (approx. width ≥ 3 fingers, hot, and/or pain)
Gut fill	0	Full, width of < 1 fist fits in the triangle of the Para-lumbar fossa.
	1	Empty, width of fist can easily fit in the Para-lumbar fossa
Decreased	0	Bright, alert, responsive
responsiveness	1	Dull, markedly depressed, or unresponsive

### Laboratory analysis

Four different blood tubes (6 ml EDTA, 6 ml heparin, 6 ml glycolytic inhibitor, and 8.5 ml serum-separator tubes) were collected per calf for each time-point. EDTA tubes were refrigerated and transported to Teagasc Grange (all IRE samples; Dunsany, Ireland) or Rimondia (all INT and NLD samples; Elspeet, The Netherlands) for haematological analysis (haematocrit and white blood cell, neutrophil, lymphocyte and monocyte counts). Haematological analysis at Teagasc Grange was performed using the Advia 2120 system (Bayer, AG). Rimondia used fluorescence flow cytometry (XT-1800i, Sysmex Europe GmbH, Germany) for haematological analyses. All haematological analyses were performed within 48 h of collection.

All remaining samples were spun at 3000 rpm for 10 min (serum-separator tubes), or 15 min (heparin & glycolytic inhibitor), decanted into serum tubes, and stored in a freezer (-20 °C). Upon completion of the trial, all serum tubes were transported to Teagasc Grange (Dunsany, Ireland). Analysis of biochemistry (glucose, beta hydroxy-butyrate (BHB), non-esterified fatty acids (NEFA), sodium, blood urea, total protein, L-lactate, creatine kinase (CK), haptoglobin) was performed using a colourmetric AU 480 chemistry analyser (Beckman Coulter, USA). Analysis of magnesium, potassium, and chloride was performed using the ISE AU 480 chemistry analyser (Beckman Coulter, USA). A commercial ELISA was used to analyse cortisol (Enzo AD1-901-071, Brussels, Belgium), serum amyloid-A (SAA) (Tri Delta TP802, Maynooth, Ireland), immunoglobulin-A and Immunoglobulin-G (Eagle BGG69-KOI, Amherst, NH, USA), and immunoglobulin-M (Eagle BCM61-KOI, Amherst, NH, USA).

### Statistical analysis

A preliminary review of the data identified negative values for some blood variables (4 records for BHB and 2 records for NEFA), which were deemed a fault of laboratory analysis and thus removed. Additionally, 12 out of 508 values for sodium, deemed unreliably low and inconsistent with clinical signs, were removed from the dataset, along with corresponding potassium, chloride, and strong-ion difference values which were similarly affected and removed. For one calf, the pre-transport weight was deemed to have been recorded in error and removed from the dataset.

SAS on Demand (SAS Statistics, 2021) was used for all statistical analyses. Two models were used for arrival (model 1) and post-arrival (model 2: repeated measures) data due to differences in management systems post arrival which may have pre-emptively impacted arrival data when used in a single model. For both model 1 and 2, blood variables were analysed using a linear mixed model (SAS Procedure PROC MIXED) to assess differences between the three groups and thereby assess the impact of transport time (long: INT, short: IRE and NLD) and fasting duration (long: INT and NLD, short: IRE) on the physiological status and health of calves. Notwithstanding cofactors, a difference in INT calves compared to IRE and NLD calves would be due to transport duration whereas a difference in INT and NLD calves compared to IRE calves would be due to fasting. Normality tests were performed on residuals, and data that failed normality tests upon initial analysis were analysed using a generalized linear mixed model (SAS procedure PROC GLIMMIX) using a lognormal distribution (BHB, NEFA, magnesium, blood urea, CK, lactate, cortisol, white blood cell count, neutrophil count, monocyte count, haptoglobin, immunoglobulin-G (IGG), immunoglobulin-A (IGA), and immunoglobulin-M (IGM)), as a square-root transformed variable (lymphocyte count, SAA), or using a binary distribution (eye discharge and navel inflammation). Covariance structure was decided based on the AIC fit statistic (ARH [1], AR [1], CSH, or Unstructured). Six health score variables (nose discharge, altered ear position, coughing, decreased responsiveness, skin tent > 1s, and gut fill) failed to converge in initial models, therefore a third model (Model 3) was employed as a minimalist model to assess whether there were any effects of treatment group and time on these clinical health scores, this model used a binary distribution.

Full details on the models are as follows:

$$\eqalign{{\rm{Model}}\,{\rm{1: }} & {\rm{ \mu + Grou}}{{\rm{p}}_{\rm{i}}}{\rm{ + Bree}}{{\rm{d}}_{\rm{j}}}{\rm{ + Se}}{{\rm{x}}_{\rm{k}}} \cr & {\rm{ + Journe}}{{\rm{y}}_{\rm{l}}}{\rm{ + Ag}}{{\rm{e}}_{\rm{m}}}{\rm{ + Weigh}}{{\rm{t}}_{\rm{n}}} \cr}$$

Where µ is the population mean, and Group_i_ (i = IRE, INT), Breed_j_ (j = HF, HF X beef cross), Sex_k_ (k = Male, Female), and Journey_l_ (l = Transported in April or May 2022) are fixed effects. Age_m_ (m = age at assembly centre: 14 d to 44 d), and Weight_n_ (n = Weight at arrival: 36.6 kg to 74.5 kg, or weight at dairy farm/mart: 39.5 kg to 75k g (∆ Body Weight at arrival only)) are included as continuous covariates.

$$\eqalign{{\rm{Model}}\,{\rm{2}} & {\rm{:}}\,{\rm{\mu + Tim}}{{\rm{e}}_{\rm{i}}}{\rm{ + Grou}}{{\rm{p}}_{\rm{j}}}{\rm{ + Bree}}{{\rm{d}}_{\rm{k}}}{\rm{ + Se}}{{\rm{x}}_{\rm{l}}}{\rm{ + Journe}}{{\rm{y}}_{\rm{m}}} \cr & {\rm{ + Ag}}{{\rm{e}}_{\rm{n}}}{\rm{ + Weigh}}{{\rm{t}}_{\rm{o}}}{\rm{ + }}\left( {{\rm{Tim}}{{\rm{e}}_{\rm{i}}}{\rm{x Grou}}{{\rm{p}}_{\rm{j}}}} \right) \cr}$$

Where µ is the population mean, and Time_i_ (i = 1 week, 3 week, or for health scores: i = 1, 3, 7, 8, and 20 days post arrival), Group_j_ (j = IRE, INT, NLD), Breed_k_ (k = HF, HF X beef) and Sex_l_ (l = Male, Female) and Journey_m_ (m = Transported in April or May 2022) are fixed effects. Age_m_ (m = age at assembly centre: 14 d to 44 d), and Weight_n_ (n = Weight at arrival: 36.6 kg to 74.5 kg) are included as continuous covariates.

$${\rm{Model}}\,{\rm{3:\mu + Tim}}{{\rm{e}}_{\rm{i}}}{\rm{ + Grou}}{{\rm{p}}_{\rm{j}}}$$

## Results

### Overview of transport groups

The final arrangements of each group is presented in Table 2 and includes distributions of calves across transport cohorts, sexes, breeds, and means and ranges of arrival ages and weights.

**Table 2:** Distribution of calves across groups (IRE, INT, NLD) and transport cohorts (C1, C2), including the distribution of calves across sexes (male, female), breeds (HF, HFxBEEF), and means and ranges for arrival ages and weights. Values represent number of calves

		C1				C2		
		IRE	INT	NLD		IRE	INT	NLD
Sex	Male	10	36	30		6	20	/
	Female	/	/	/		4	9	/
Breed	HF	3	5	30		10	10	/
	HFxBEEF	7	31	/		/	19	/
Age (d)	mean (range)	28.5 (18–41)	29.9 (16–42)	17.7 (14–24)		31.1 (24–35)	18.6 (17–36)	/
Weight on arrival (kg)	mean (range)	55.9 (39–74)	52.6 (40–63)	42.5 (36–48)		55.6 (49–59)	54.6 (41–71)	/

All INT and NLD calves received antibiotic treatment shortly after arrival (Tilmicosin: 1 cc I.M.), and received an additional two courses of metaphylactic batch antibiotics in week 1 and week 2 post arrival at the veal farm (Doxycycline: 0.03 g per feed in milk twice a day for three and five days respectively). One NLD calf received a dose of Florfenicol (8 cc I.M.) 17 days post arrival due to symptoms of respiratory disease. Within the IRE group, a respiratory disease outbreak (pathogen undetermined) two days post arrival necessitated an antibiotic treatment for four calves (Amoxicillin, 5 ml I.M, once daily for five days). Further to this, a subsequent respiratory disease outbreak 10 days post arrival, an additional 6 IRE calves (10 in total) received antibiotics (seven given a single dose of a long-acting Oxytetracycline: 1 ml/10 kg body weight and three given a single dose of Oxytetracycline: 2 ml/10 kg body weight I.M., once a day for five days). No calf in any of the transport groups died during the transport or for three weeks thereafter.

### Effect of transport duration and fasting duration on blood variables

#### Blood variables on arrival

**Fig. 2:**
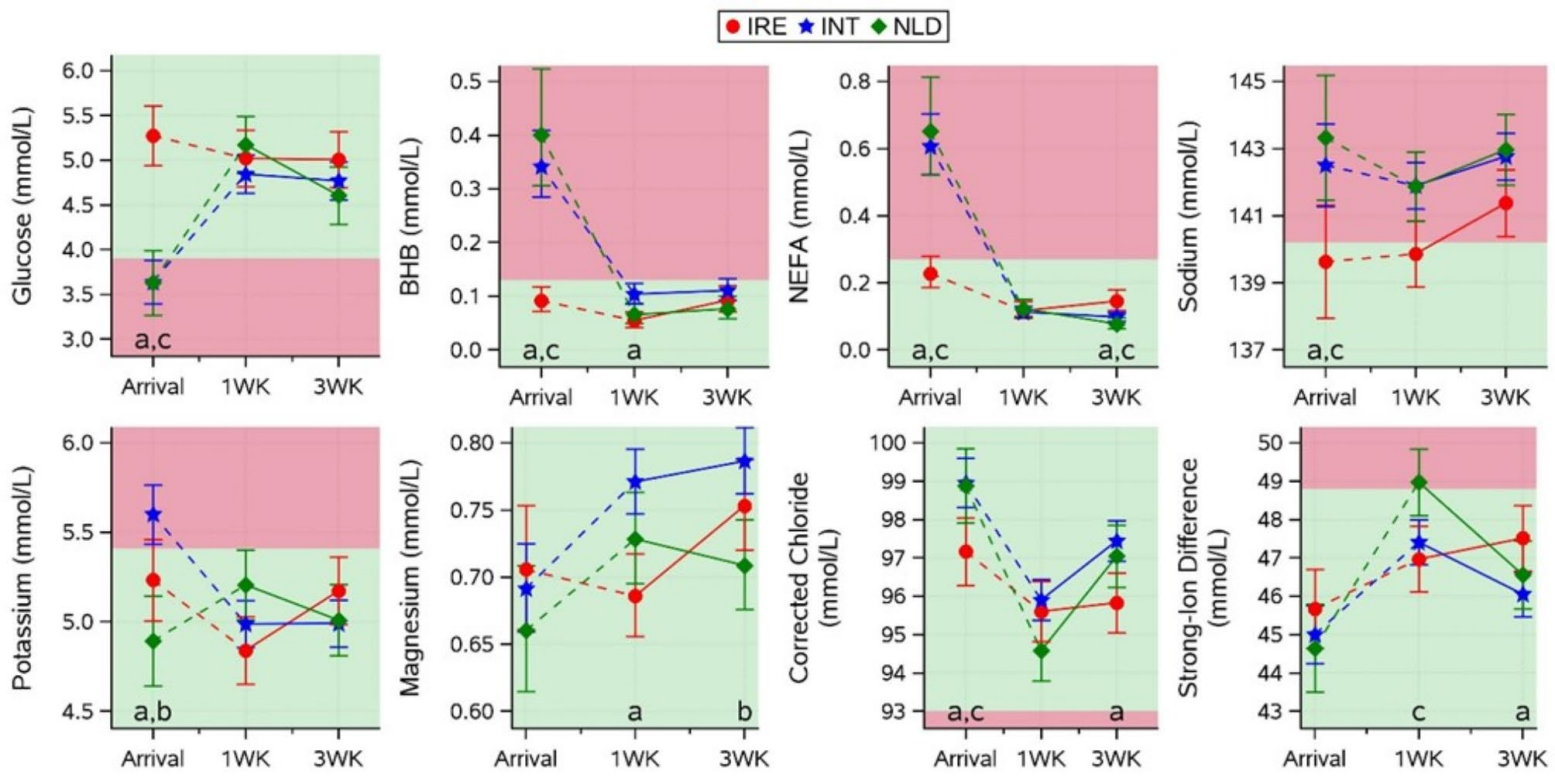
Effect of transport group (IRE, INT, NLD) on blood variables related to energy balance and electrolytes (glucose, BHB, NEFA, sodium, potassium, magnesium, corrected chloride and strong-ion difference (mean ± CI)) on arrival and the interaction of group and time (1WK: 1 week post arrival, 3WK: 3 week post arrival) on the recovery of these blood variables. Values for arrival and subsequent recovery were analysed in 2 different statistical models. Subscripts highlight significant (*p* < 0.05) differences between IRE and INT (a), between INT and NLD (b), or between IRE and NLD (c) calves within a timepoint. Shaded areas represent areas outside (red/dark) or inside (green/light) reference limits (based on previous research [22–24])

Calculated blood values and statistically significant differences for electrolytes and blood variables related to energy balance for all groups on arrival are presented in Fig. 2. On arrival at their respective destination farms (Ireland/The Netherlands), IRE calves had higher glucose than INT and NLD calves (5.3 vs. 3.6 and 3.6 mmol/L respectively; both *p* < 0.01), while BHB and NEFA were lower for IRE calves than for INT and NLD calves (BHB: 0.09 vs. 0.34 and 0.40 mmol/L; NEFA: 0.23 vs. 0.61 and 0.65 mmol/L respectively; *p* < 0.01). For all three energy-balance related variables, values for the IRE group fell within normal reference limits on arrival, but values for INT and NLD groups were below (glucose: 3.9–8.4 mmol/L) or above (BHB: 0–0.13 mmol/L [22] and NEFA: 0.04–0.27 mmol/L [23]) normal reference limits. Sodium was lower for IRE calves than for INT and NLD calves (139.6 vs. 142.5 and 143.3 mmol/L; *p* < 0.05); IRE calves were within the reference limits (133.3–140.2 mmol/L [24]) while INT and NLD calves had values above the reference limit on arrival. Additionally, corrected chloride was lower for IRE (97.3 mmol/L) than for INT and NLD calves (99.0 and 98.9 mmol/L respectively) on arrival (both *p* < 0.05), although all groups had chloride levels within the reference limits (93–101 mmol/L [24]).

**Fig. 3:**
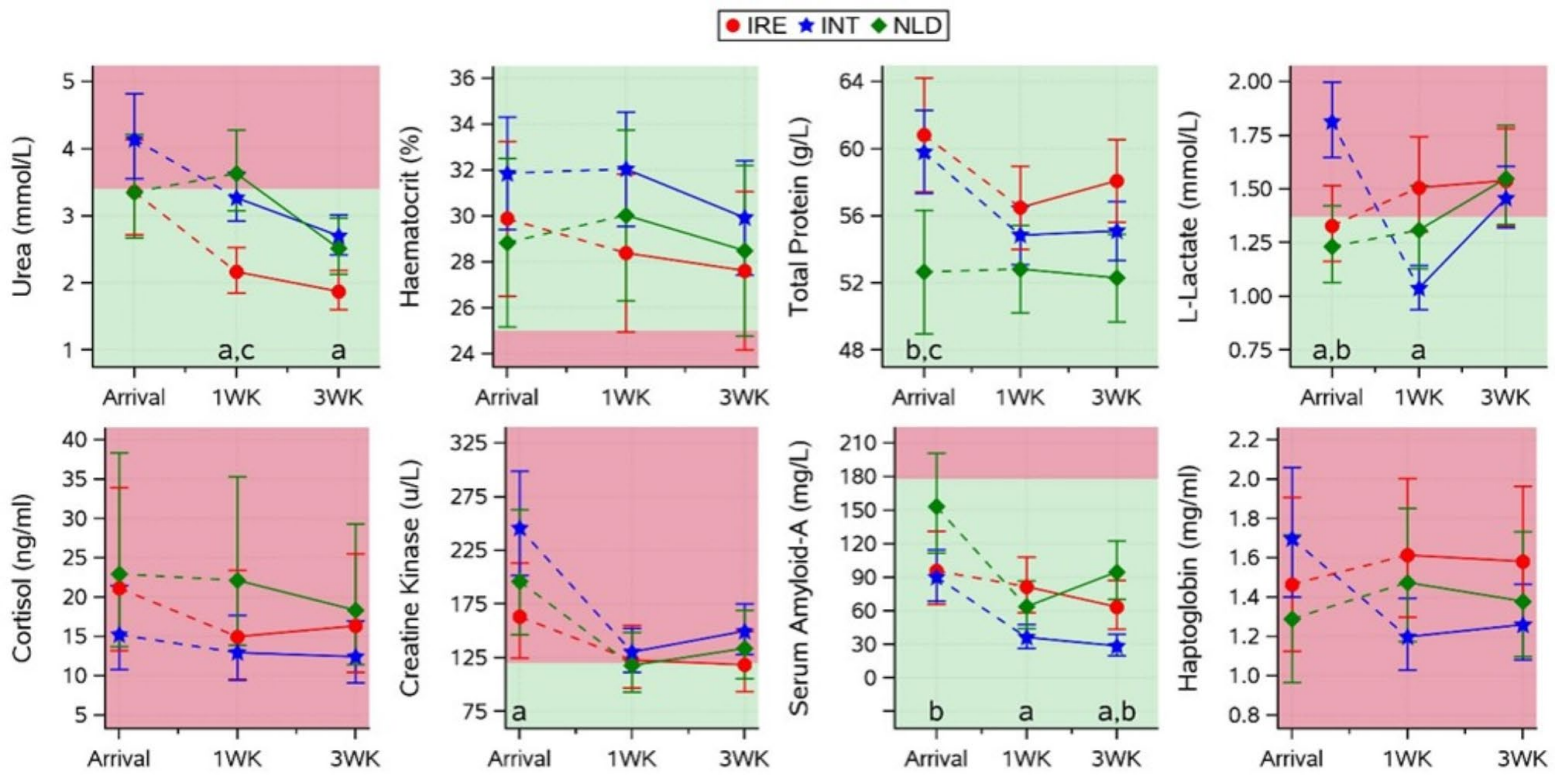
Effect of transport group (IRE, INT, NLD) on urea, haematocrit, total protein, L-lactate, cortisol, creatine kinase, SAA, and haptoglobin (mean ± CI)) on arrival and the interaction of group and time (1WK: 1 week post arrival, 3WK: 3 week post arrival) on the recovery of these blood variables. Values for arrival and subsequent recovery were analysed in 2 different statistical models. Subscripts highlight significant (*p* < 0.05) differences between IRE and INT (a), between INT and NLD (b), or between IRE and NLD (c) calves within a timepoint. Shaded areas represent areas outside (red/dark) or inside (green/light) reference limits (based on previous research [22–27])

Calculated blood values and statistically significant differences for urea, haematocrit, total protein, L-lactate, cortisol, creatine kinase, SAA, and haptoglobin for all groups on arrival are presented in Fig. 3. On arrival, blood urea values tended to be higher for INT than for IRE calves (4.14 vs. 3.35 mmol/L; *p* = 0.08), and INT were the only group presenting with blood urea values above the reference limit (0.4–3.4 mmol/L [22]). Total protein was lower for NLD calves than for IRE and INT calves (52.6 vs. 60.8 and 59.8 g/L; *p* < 0.01), but was within reference limits for all groups (40–90 g/L [25]). Lactate was higher for INT than for IRE and NLD calves (1.81 vs. 1.33 and 1.23 mmol/L respectively; *p* < 0.01), and was within reference limits (0.33–1.37 mmol/L [26]) for IRE and NLD calves but not for INT calves. SAA did not show differences between IRE (90.2 mg/L) and other groups but was lower for INT than for NLD calves (95.6 and 153.1 mg/L respectively; *p* = 0.03), SAA was considered to be within the reference limits (< 178 mg/L [27]) for all groups.

**Fig. 4:**
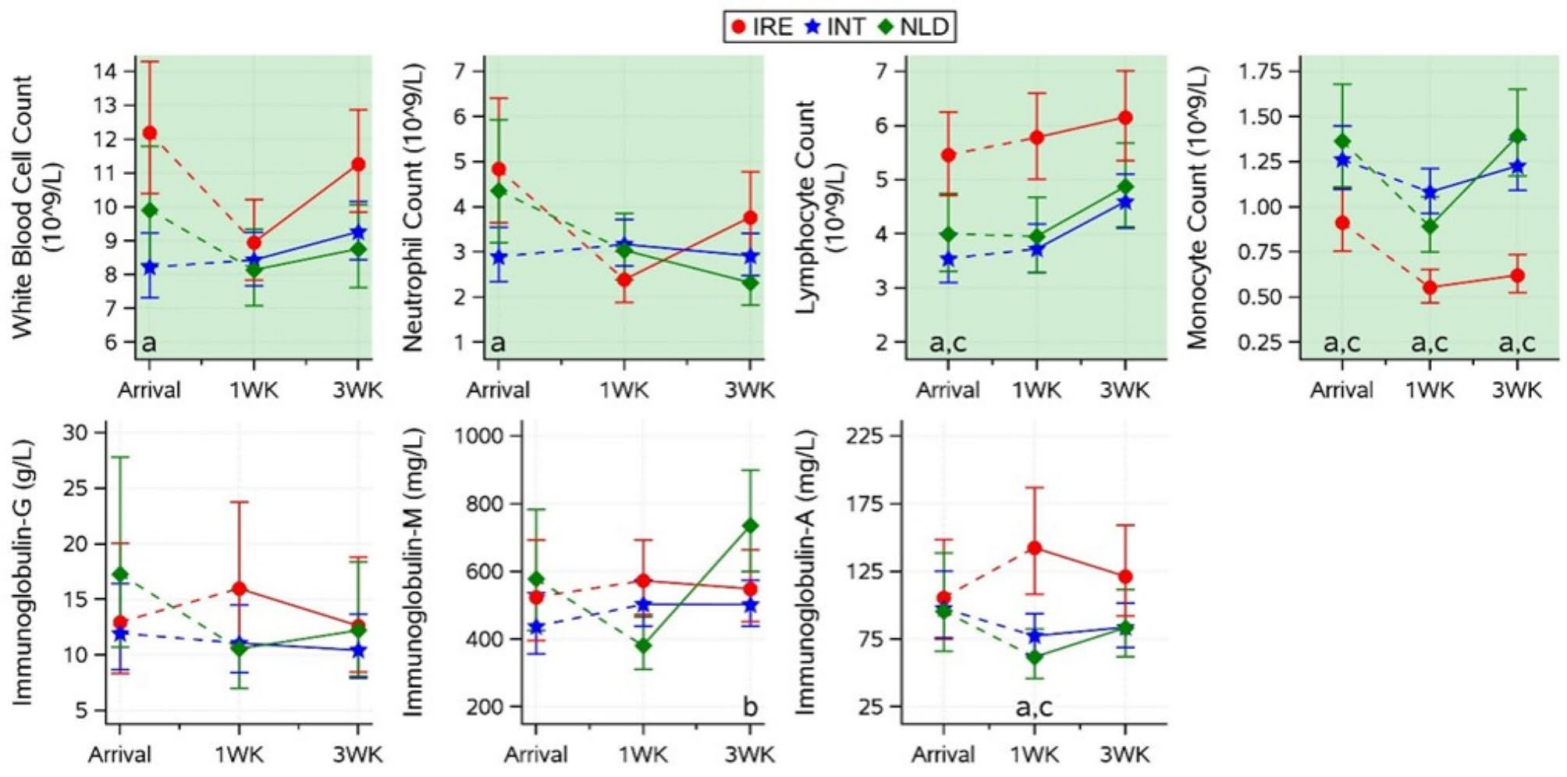
Effect of transport group (IRE, INT, NLD) on immune cell subsets (white blood cell, neutrophil lymphocyte, and monocyte counts) and immunoglobulins (A, G, and M) (mean ± CI)) on arrival and the interaction of group and time (1WK: 1 week post arrival, 3WK: 3 week post arrival) on the recovery of these blood variables. Values for arrival and subsequent recovery were analysed in 2 different statistical models. Subscripts highlight significant (*p* < 0.05) differences between IRE and INT (a), between INT and NLD (b), or between IRE and NLD (c) calves within a timepoint. Shaded areas represent areas outside (red/dark) or inside (green/light) reference limits (based on previous research [25]) except for immunoglobulins G, M, and A, for which no reference limits were available

Calculated blood values and statistically significant differences for immune cell subsets and immunoglobulins for all groups on arrival are presented in Fig. 4. White blood cell and neutrophil counts were higher for IRE (12.20 and 4.84 10^9^/L respectively) than for INT calves (8.21 and 2.89 10^9^/L respectively; *p* < 0.01). IRE calves showed higher lymphocyte count and lower monocyte count (5.46 and 0.91 10^9^/L respectively) than their INT (3.54 and 1.26 10^9^/L respectively) and NLD (4.00 and 1.37 10^9^/L respectively) counterparts (all *p* < 0.05). All immune cell subsets were within reference limits for all groups (white blood cell: 2.55–23.73 10^9^/L; neutrophil: 0.39–15.78 10^9^/L; lymphocyte: 0.17–9.45 10^9^/L; monocyte: 0.01–2.11 10^9^/L [25]). No differences were found in IgA, IgG, and IgM, for which no reference limits were available.

### Blood variables 1- and 3-weeks post arrival

Estimated blood values and significant differences for all groups at 1 and 3 weeks post arrival are also presented in Figs. 2 and 3, and Fig. 4. BHB concentrations were slightly lower for IRE calves compared to INT calves 1 week post arrival (0.05 vs. 0.10 mmol/L; both *p* < 0.01) while NEFA was higher for IRE compared to INT and NLD calves 3 weeks post arrival (0.14 vs. 0.10 and 0.08 mmol/L; both *p* < 0.01), despite mean glucose, BHB, and NEFA values falling within the reference limits for all groups at 1 and 3 weeks post arrival.

Blood urea showed a decreasing pattern between arrival and 3 weeks post arrival, at 1 week post arrival, blood urea was lower for IRE than for INT and NLD calves (2.2 vs. 3.3 and 3.6 mmol/L; both *p* < 0.01), by 3 weeks post arrival all groups had mean urea values within reference limits. Other dehydration indicators such as haematocrit and total protein did not differ between groups 1 and 3 weeks post arrival and were within reference limits throughout the study. Lactate was higher for IRE than for INT calves 1 week post arrival (1.5 vs. 1.0 mmol/L; *p* < 0.01), but levels were similar to IRE and NLD calves by 3 weeks post arrival, where all groups had mean lactate levels above the upper reference limit of 1.37 mmol/L [26]. Cortisol and CK did not differ between groups 1 and 3 weeks post arrival; cortisol levels were consistently above reference limits for all groups while mean CK levels were close to upper reference limits one and three weeks post arrival [22]. Inflammatory marker SAA was higher for IRE than for INT calves at 1 week post arrival (81.5 vs. 36.1 mg/L; *p* < 0.01) and 3 weeks post arrival (63.4 vs. 28.6 mg/L; *p* < 0.01). SAA was also higher for IRE than for NLD calves 3 weeks post arrival (63.4 vs. 28.6 mg/L; *p* < 0.01), even though SAA was consistently within reference limits [27] for all groups. Haptoglobin showed large variations within groups and lacked interactions between groups 1 and 3 weeks post arrival (*p* = 0.41), and was consistently above the upper reference limit [23] for all groups.

At 3 weeks post arrival, IRE calves tended to have a higher white blood cell count (vs. INT, 11.3 vs. 9.3 10^9^/L; *p* = 0.04) and neutrophil count (vs. NLD, 3.76 vs. 2.32 10^9^/L; *p* = 0.09). Lymphocyte count did not show an interaction between group and time (*p* = 0.14), but at both 1 and 3 weeks post arrival monocyte count was lower for IRE (0.55 and 0.89 10^9^/L) than for INT (1.08 and 1.23 10^9^/L) and NLD (0.89 and 1.39 10^9^/L) calves (all *p* < 0.01). Immune cells white blood cell, neutrophil, lymphocyte and monocyte counts were consistently within reference limits for all groups [25]. Changes in immunoglobulins were small, except for a peak in IgA concentrations for IRE compared to INT and NLD calves 1 week post arrival (142.4 vs. 77.3 and 61.6 mg/L; both *p* < 0.01), and a peak in IgM for NLD calves 3 weeks post arrival, which was higher than that of INT calves (735 vs. 502 mg/L; *p* = 0.04). Reference limits were not available for immunoglobulins.

### Effect of transport type and fasting on clinical health and body weight

Proportions of various clinical health scores at arrival and the effect of group are presented in Table 3. Eye discharge, skin tent, and navel inflammation did not differ between groups (all *p* > 0.1), and models for other health score variables did not converge due to low proportions of health scores in some groups.

**Fig. 5:**
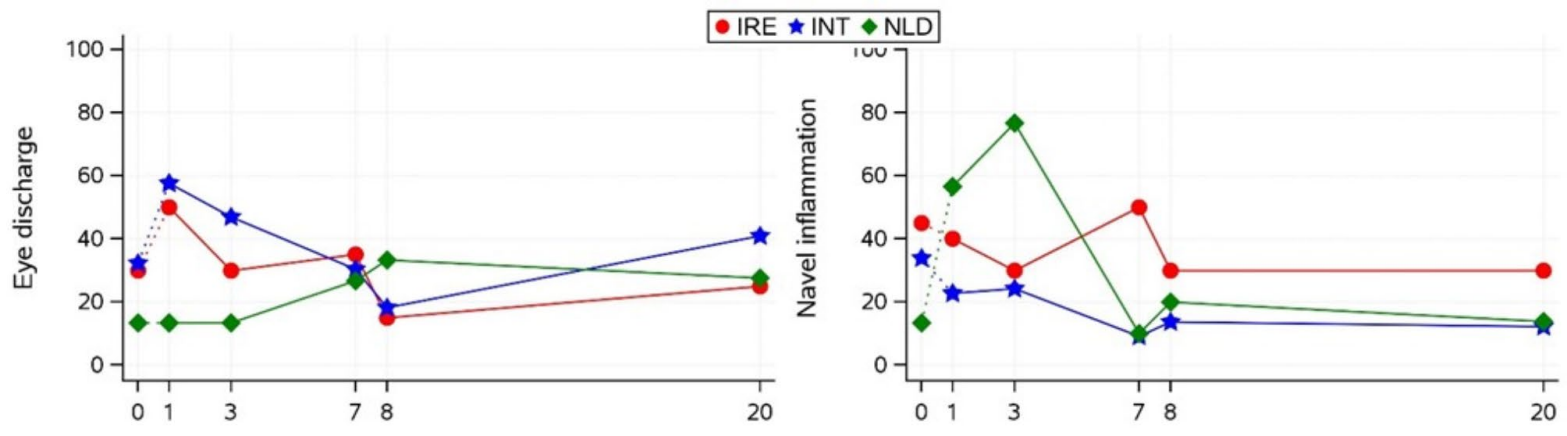
Effect of group (IRE, INT, NLD) on eye discharge and navel inflammation (proportion of calves affected) on arrival and 1, 3, 7, 8, and 20 days post-arrival. Values for arrival and subsequent recovery were analysed in 2 different statistical models. No significant differences were present between groups within any timepoint

Proportions of various clinical health scores post arrival and effects of group, time and the interaction between group and time are presented in Table 4. Eye discharge and navel inflammation alone showed interactions between group and time, as shown in Fig. 5, while no significant differences in eye discharge or navel inflammation were found between groups on any day. However, the proportion of calves with eye discharge tended to be larger for INT than for NLD calves 1 day post arrival (0.57 vs. 0.13; *p* = 0.07). The proportion of calves with navel discharge tended to be smaller for INT than for NLD calves 3 days post arrival (0.24 vs. 0.76; *p* = 0.09) and tended to be larger for IRE than for INT calves 7 days post arrival (0.50 vs. 0.09 calves; *p* = 0.09).

**Table 3:** Proportions of calves for IRE, INT, and NLD groups showing clinical health scores on arrival at the destination farm and effects (p-values) of treatment groups on clinical health score variables

Variable	Group			*p*-value
	IRE	INT	NLD	
Eye discharge	0.30	0.32	0.12	0.12
Nose discharge	0.00	0.17	0.00	-
Altered ear position	0.00	0.05	0.03	-
Coughing	0.00	0.03	0.00	-
Decreased responsiveness	0.00	0.05	0.00	-
Skin tent > 1s	0.20	0.42	0.63	0.26
Gut fill	0.25	0.31	0.00	-
Navel Inflammation	0.45	0.34	0.13	0.70

‘-‘= model did not converge

**Table 4:** Proportions of calves for IRE, INT, and NLD groups showing clinical health scores and effects (p-values) of group and time (1, 3, 7, 8, and 20d post arrival), and interactions between group and time on clinical health score variables

Variable	Group				*p*-value		
	IRE	INT	NLD		Group	Time	Group * Time
Eye discharge	0.31	0.39	0.23		0.08	0.17	0.02
Nose discharge	0.17^a^	0.17^a^	0.05^b^		< 0.01	< 0.01	-
Altered ear position	0.03^a^	0.04^a^	0.19^b^		< 0.01	< 0.01	-
Coughing	0.15	0.05	0.00		-	-	-
Decreased responsiveness	0.20	0.02	0.04		-	-	-
Skin tent > 1s	0.08^a^	0.06^a^	0.16^b^		< 0.01	< 0.01	-
Gut fill	0.06	0.28	0.00		-	-	-
Navel Inflammation	0.36	0.16	0.36		0.02	< 0.01	< 0.01

‘-‘= model did not converge, mean values without a common superscript (^a, b^) differ significantly (p < 0.05)

The proportion of calves with nose discharge was smaller for NLD than for INT and IRE calves (both *p* < 0.01), nose discharge was found less frequently on day 20 than on day 1 (*p* < 0.01), day 3 (*p* = 0.03), day 7 (*p* = 0.03), and day 8 (*p* = 0.01). Altered ear position was found more frequently in NLD than in INT and IRE calves (*p* < 0.01 and *p* = 0.01 respectively) and was more frequent on day 7 than on day 8 (*p* = 0.02) and day 20 (*p* < 0.01). A skin tent > 1s was found more frequently in NLD than in INT or IRE calves (both *p* < 0.01), and tended to be found more frequently on day 1 than day 8 post arrival (*p* = 0.05). Models for coughing, decreased responsiveness and gut fill did not converge due to low frequencies of these health scores observed, though it must be noted that there was a large proportion of IRE calves presenting with coughing relative to other groups (0.15 in IRE vs. 0.05 and 0.00 in INT and NLD), while the INT group numerically had a much larger proportion of calves with an empty gut fill compared to other groups during the recovery period (0.28 in INT vs. 0.06 and 0.00 in IRE and NLD).

Changes in body weight between the farm/mart of origin and arrival on the veal farm, and between arrival on the veal farm and three weeks post arrival are presented in Table 5. For IRE and INT groups, 65% and 94% of calves lost weight during their journeys’ respectively, INT calves lost significantly more weight during transport than IRE calves (*p* < 0.01), amounting to an average loss of an additional 1.79 kg more than the IRE group (4.2% and 1.3% of body weight lost during the journey respectively). During recovery, INT and NLD calves gained similar weight which was significantly less than that gained by the IRE calves (*p* < 0.01), amounting to an average overall additional gain of 3.7 kg relative to the INT group, over the same 21-day period.

**Table 5:** Effect of group on body weight loss and gain (in kg; mean ± standard error) between the dairy farm/mart of origin and arrival on the veal farm (∆ farm of origin – arrival), and between arrival on the veal farm and three weeks post arrival on the veal farm (∆ arrival – 3 week)

	Group			*p*-value
	IRE	INT	NLD	
Arrival Weight	55.7 (± 1.689)	53.5 (± 0.838)	42.46 (± 0.560)	
∆ Farm of origin - arrival	− 0.71 (± 0.402) ^a^	− 2.37 (± 0.296) ^b^	-	< 0.01
∆ Arrival – 3 week	+ 10.7 (± 0.729) ^a^	+ 7.00 (± 0.533) ^b^	+ 6.69 (± 0.826) ^b^	< 0.01

Mean values without a common superscript (^a, b^) differ significantly (*p* < 0.05)

## Discussion

Live transport, by its nature, is a variable event with combined periods of moving and waiting at staging posts, which are often repeated events. A calf’s negative experiences during transport are a summation of psychological and physical strain, the need to constantly adjust to the truck’s unsteady movements, and feed deprivation culminating in an altered physiological status and loss of homeostasis. The aim of this observational study was to determine how transport and fasting durations impacted the physiology and health of transported calves in three transport groups. The extensive transport and fasting durations experienced by calves in this study and resulting changes in their physiological status emphasise the need for changes in the management of calves before, during, and after transport to mitigate the negative effects of transport.

### Overview of transport groups

Typically, Irish calves heading for Irish rearing farms are transported for no more than 12 h, whereas Irish calves headed for veal farms in The Netherlands can experience total journey (transport and resting) times of 79 h [15]. Dutch born calves destined for veal farms are typically transported for short distances to collection centres where they remain for a few hours (although average durations are unknown but may legally last up to 30 h; NL regulation #WJZ/18205482) and are usually fed milk replacer once, after which they are transported for one to four hours to a veal farm [28]. In all these systems calves are commonly fed milk (replacer) prior to leaving the farm of origin and again upon arrival at their destination. In the case of a resting period at an assembly centre or lairage (internationally transported and Dutch calves) they are usually fed milk replacer (commonly 2 L) once at each assembly centre/lairage.

In the current study, the IRE group experienced a journey time of approximately 29 h, transport of up to 13 h and a single feed withdrawal time of 11 h prior to arrival, which could be regarded, in relative terms of duration, as the lowest impact of all three groups. Similar to the IRE group, the NLD group experienced a journey time of 28 h and transport of 4 h, however they had a prolonged feed withdrawal time, estimated at > 18 h, a period suggested by the owners as being atypically long compared to normal industry practice (personal communication), although no public records state average journey times of Dutch calves heading for veal farms. In relative terms, the INT group went through the most challenging experience during their journey, with a prolonged journey time of approximately 79 h, repeated transport totalling 40 h (sea and road) and prolonged repeated feed withdrawal periods of 27–30 and 24–28 h.

Analysis of the blood variables for all groups during transport demonstrated that IRE calves displayed three (out of twenty-three) variables outside reference limits, namely cortisol, creatine kinase, and haptoglobin. The NLD group displayed seven (out of twenty-three) variables outside the reference limits upon arrival, namely the same variables as the IRE group, in addition to glucose, BHB, NEFA, and sodium. For the INT group, ten (out of twenty-three) variables were outside reference limits upon arrival, namely the same variables as the IRE and NLD groups, in addition to potassium, blood urea, and lactate. It was clear that the number of variables outside of reference ranges progressively increased in groups with increased welfare impacts, including fasting (NLD) and fasting plus transport (INT).

### Fasting duration

Previous research has shown that energy balance is altered following long-distance transport, as evidenced by a decrease in glucose and corresponding increase in BHB and NEFA [4, 11, 12, 29]. This profile of change was evident, and pronounced, for both the INT and NLD group on arrival, both showed signs of a deteriorated energy balance on arrival, particularly relative to the IRE group, with mean values of all three energy balance related variables outside of reference limits for these two groups. The almost identical average values for these variables in the INT and NLD groups on arrival were notable (glucose: 3.64 vs. 3.63 mmol/L; BHB: 0.34 vs. 0.40 mmol/L; NEFA: 0.60 vs. 0.65 mmol/L for INT and NLD respectively), despite undergoing a different journey experience. The added factor of transport combined with prolonged fasting in the INT group did not lead to a worsening of the profile of these three energy-based variables, suggesting that fasting in itself may be as detrimental to the energy balance of calves as combined fasting and transport. Calves were fed low volumes of milk replacer (2 to 3 L depending on the location) before, during, and after transport, which, based on energy balance and hydration indicators, was clearly inadequate for their nutritional needs. The low volume of milk replacer provided, in conjunction with extensive fasting times, negatively impacted the calves’ energy balance. Increasing both the volume and frequency of milk replacer feedings will aid in the ability of these calves to cope with transport.

Dehydration did not appear to be a standout complication in this study and was in line with prior research highlighting inconsistencies on blood electrolytes, total protein and blood urea during long transport and fasting [3, 12, 30–32]. In the present study, magnesium, chloride (corrected), SID and haematocrit were comparable across groups and within reference limits throughout.

Sodium, as the principal electrolyte in the extracellular fluid, is most sensitive to small fluid volume changes which could be anticipated by the prolonged feed withdrawal and associated reduction in water intake in these two groups. Both INT and NLD groups had sodium levels that were elevated to above normal reference levels (133.3–140.2 mmol/L; [24]) on arrival, which was not observed in IRE calves, and a possible sign of fluid volume changes in these fasted calves, however, average values were not in the hypernatraemic range (sodium > 145 mmol/L [33]) in these groups, and therefore could not be used to classify severe dehydration. Blood urea values were not significantly different on arrival despite almost three times as many calves in the INT group having blood urea values outside of the reference limits relative to the IRE group (68% versus 25% of calves; 50% in the case of the NLD group). Even though levels of blood urea decreased for all groups after arrival, this change was slower and more inconsistent for INT and NLD groups suggesting again an effect of fasting rather than fasting combined with transport. Previous transport research highlighted a similar gradual decrease in blood urea levels between arrival (> 6 mmol/L) and 3 weeks post arrival (approx. 2.8 mmol/L) in calves transported for long distances with increased fasting time [11]. Dehydrated calves typically show enophthalmos and a delayed skin tent response [3], but only the NLD group showed this effect on arrival (albeit not significantly different from IRE nor INT groups), whereas other dehydration variables, such as blood urea, were higher for the INT group, it is unclear what caused these inconsistencies. This overall disparity amongst dehydration variables in this study and other publications may also point to poor sensitivity of these markers, a factor that requires closer scrutiny in future transport research.

Loss of weight is a frequently observed outcome of transport of calves emanating primarily from the imposed fasting [29, 34]. Regular feeding is essential to maintain body weight, consequently the INT group in this trial were greatest impacted; 94% of INT calves lost weight during the journey while 65% of IRE calves lost weight during their journey. However, even INT calves lost less weight than reported previously; weight losses of calves during transport commonly range from 6.5 to 7.5%, and lower arrival weights are related to higher morbidity incidence [4, 29, 35]. INT and NLD calves gained less weight than IRE calves during the three week period post arrival, a difference which could be attributed to different feeding regimes offered post arrival (2 L versus 3 L milk replacer twice a day for the for the INT/NLD and IRE groups, respectively).

Transport-associated bovine respiratory disease (BRD), or shipping fever, is the most likely cause of compromised immunity during transport and is likely to be enabled through a combination of exposure to increased infectious load during the journey and physical strain derived from the journey experience itself [8, 36]. In this study, the cellular immunity profile of the IRE group on arrival is, on average, in line with reference expectations for neutrophils, lymphocytes and monocytes. In relative terms compared to the IRE group, the INT group arrival data displayed a profile of monocytosis and lymphopenia, both key elements of a stress leukogram, and neutropenia (potentially consumptive due to viral exposure and infection, although commonly rises with a stress leukogram). All immunological variables were different in INT compared to IRE (white blood cell and neutrophil counts) or in INT compared to IRE and NLD groups (lymphocyte and monocyte counts) on arrival, indicating INT calves experienced an impacted immune system after transport. Apart from neutrophil values on arrival, the NLD group’s cellular immune profile is comparable to the INT group throughout the analysis period. It was only those two groups who shared prolonged feed withdrawal that displayed a comparable physiological decline in immune cell numbers. It could be argued, therefore, that a key driver of this relative decline is prolonged fasting and the strain of hunger and thirst rather than long distance transport alone.

The blanket treatment of the INT and NLD groups with antibiotics shortly after arrival most likely circumvented the onset of BRD in these groups facilitating a recovery in lymphocyte numbers, but evidently not monocytes. The high risk of morbidity post-transport was evident in this study, highlighted by the fact that the one group without blanket antibiotic treatments and the least impactful journey (i.e. IRE calves) succumbed to infection. The influence of local husbandry factors contributing to disease outbreak in this group cannot be dismissed, however, the strain of mixing young animals combined with transport and fasting, must be considered the major risk factor in this case.

### Transport duration

Blood L-lactate and CK were employed as indicators of muscle fatigue and damage, respectively. The anticipated rise in these variables in the INT group above the other two was observed, as reported in a previous publication on the same group of calves [15], where CK levels were higher after the end of the journey than at the start or at resting points during the journey. The rise in lactate and CK in INT compared to other groups was obvious, rising to higher concentrations in INT than in IRE and/or NLD calves on arrival. However, despite a relative reduced transport duration, the CK levels in the IRE and NLD groups were also above the upper reference limit. In contrast, unlike the INT group, L-lactate levels were within range for these groups. Combined, this profile suggests that L-lactate appears to be a more sensitive indicator of transport duration relative to CK, most likely reflecting prolonged use of muscle tissue to proprioceptively counteract truck movements [6], as demonstrated in the INT group. While a concurrent mild dehydration in this group may falsely elevate these variables to some degree, these blood variable changes highlight the impact of long-distance transport on young calves. The recovery profile in the three groups for these variables was not consistent. The reduction in CK levels across all groups over the first week was followed by a small increase in the INT and NLD groups. In the INT group, L-lactate fell sharply by 1 week, but increased slightly for the other two groups. The reasons for these changes were not obvious.

Blood potassium was initially included as an electrolyte and indicator of fluid volume changes, but levels of potassium between groups did not follow this expectation. Potassium levels were unremarkable apart from the arrival timepoint, where values were higher and marginally outside of the upper reference limit in the INT group, a rise which could be explained by the increase in muscle cell trauma due to transport and correlate with increased CK and L-lactate levels, and changes therefore were likely due to transport duration, rather than dehydration or fasting.

### Other factors

SAA was lower in the INT group than in the NLD group at arrival, contrary to expectation; SAA has several physiological roles [37], and from a research perspective is commonly used as a biomarker of the primordial acute phase response and as a diagnostic tool for BRD [38]. An indirect benefit of antibiotics offered to the INT and NLD groups, leading to a reduction in infection, may also facilitate or contribute to this lower level. While some calves in the IRE group succumbed to infection within 2 days of arrival, the average for this group was within range. A review of blood SAA in ill IRE calves revealed values marginally above the upper reference limit (178 mg/L; [27]) and perhaps increasing as the infection took hold. Blood levels of haptoglobin were not significantly differentiated at any time point by the alternative journey forms, despite being outside of the reference limits in all three groups for the duration of the analysis. An increase in haptoglobin is typically expected as a result of respiratory disease, but the fact that levels were well above reference limits, which was already the case for INT calves at their point of origin in previous results [15], hints at other unknown causes.

Clinical health scores were unremarkable on arrival and differences between groups were inconsistent during recovery. Clinical signs of pneumonia include a combination of eye discharge, nose discharge, altered ear position and coughing [20], but this pattern was not seen in any group. Navel inflammation rates are more common in younger calves, such as the NLD group [35, 39], while they had similar navel infection rates as the IRE group. A loss of gut fill can also be attributed to weight loss in the first 24 h of fasting [40]. Gut fill visually was worse for INT than IRE calves, but empty gut fill was never observed for the NLD group. The smaller and leaner conformation of NLD calves compared to Irish-born calves may have hampered the functionality of this variable. Also, total protein was lower in the NLD group on arrival, a difference which could be explained by the difference in age, whereby the NLD group was made up of calves between 14 and 24 days of age while IRE and INT calves were mostly older.

### Limitations

This study encountered a number of noteworthy limitations. Firstly, calves born in The Netherlands differed from those born in Ireland; NLD calves were younger, weighed less and this cohort differed in the breakdown of their breed. Despite recording these factors and accounting for them in the statistical procedures, such factors may have had unknown basal effects on the physiology and health of these calves. Secondly, specific elements in the management of the NLD calves were unknown which limited the conclusions that were drawn from the differences between treatment groups.

Thirdly, different husbandry procedures at the Irish and Dutch destination farms, particularly relating to housing, feeding and medicinal strategies limited the comparative analysis of the post-transport recovery period. In recognition of these differences, separating the statistical analysis into transport up to arrival and subsequent timepoints was necessary.

Finally, the lack of observers jointly training in completing clinical health scoring due to logistical complications may have impacted the scoring outcomes. While several external factors impacted the sensitivity of clinical health scoring variables, including the use of antibiotics and inter-observer variability, the need to detect comparable subtle differences, rather than physical health scores, suggest investigating more sensitive non-invasive continuous monitoring methods would be worthwhile. Future research focusing on the use of continuous health monitoring (such as continuous glucose monitors), continuous behaviour recording (i.e. activity sensors), and other forms of detecting respiratory disease (i.e. thoracic ultrasound scoring or radiography) would appear to be of greater benefit.

### Implications of the results for the industry

This study employed several blood and clinical health variables that would likely be altered by different journey experiences. While some variables have proven their usability, in particularly those concerned with energy balance (glucose, BHB and NEFA), sodium in relation to dehydration, and L-lactate and potassium in relation to muscle fatigue, others proved to be unchanged or insensitive to journey experiences, including the remaining electrolytes, haematocrit, total protein, SAA, haptoglobin, and all immunoglobulins included in this study. However, the changes in energy balance and dehydration in fasted calves, and muscle fatigue in calves transported for a long duration, clearly highlight aspects of impaired welfare (fasting more clearly than transport duration) that should be the focus of future research and legislative changes. Such a focus should encompass developing systems to allow for regular feeding during transport, or on setting maximum fasting times during transport between the farm of origin and destination farm, rather than looking at transport duration alone. Future changes to legislation governing calf transport should mandate regular feeding of sufficient volume and quality for calves before, during, and after transport to support their heightened energy and hydration demands.

## Conclusion

In conclusion, this study indicates that all three groups of transported calves were negatively impacted by transportation. The calves exposed to combined feed deprivation and extended transport in this study yielded deterioration in energy (glucose, BHB, NEFA), electrolyte (sodium) and immunological variables (lymphocytes and monocytes) comparable to calves exposed to feed deprivation alone. This suggests that prolonged feed deprivation is a critical risk factor in altered physiological status, perhaps more important than transport itself, which accounted for two additional impacts alone related to muscle damage and fatigue (L-lactate and potassium). All groups showed patterns of recovery post-transport, and were within reference limits for all but five variables by week 3. Based on these results, improved physiological status and health of calves during the journey could be achieved in the first instance by shortening the period of feed deprivation, which should be considered in future changes to legislation in the area of calf transport.

## Data Availability

The datasets used and/or analysed during the current study are available from the corresponding author on reasonable request.
